# Plasma Cytokine Expression Is Associated with Cardiac Morbidity in Chagas Disease

**DOI:** 10.1371/journal.pone.0087082

**Published:** 2014-03-06

**Authors:** Giovane Rodrigo Sousa, Juliana Assis Silva Gomes, Rafaelle Christine Gomes Fares, Marcos Paulo de Souza Damásio, Ana Thereza Chaves, Karine Silvestre Ferreira, Maria Carmo Pereira Nunes, Nayara Ingrid Medeiros, Vanessa Alves Azevedo Valente, Rodrigo Corrêa-Oliveira, Manoel Otávio da Costa Rocha

**Affiliations:** 1 Programa de Pós-graduação em Ciências da Saúde: Infectologia e Medicina Tropical, Faculdade de Medicina, Universidade Federal de Minas Gerais, Belo Horizonte, Minas Gerais, Brazil; 2 Departamento de Morfologia, Instituto de Ciências Biológicas, Universidade Federal de Minas Gerais, Belo Horizonte, Minas Gerais, Brazil; 3 Laboratório de Imunologia Celular e Molecular, Centro de Pesquisas René Rachou, Fundação Oswaldo Cruz, Belo Horizonte, Minas Gerais, Brazil; 4 Instituto Nacional de Ciência e Tecnologia em Doenças Tropicais, Belo Horizonte, Minas Gerais, Brazil; Albert Einstein Institute for Research and Education, Brazil

## Abstract

The expression of immune response appears to be associated with morbidity in Chagas disease. However, the studies in this field have usually employed small samples of patients and statistical analyses that do not consider the wide dispersion of cytokine production observed in these patients. The aim of this study was to evaluate the plasma cytokine levels in well-defined clinical polar groups of chagasic patients divided into categories that better reflect the wide cytokine profile and its relationship with morbidity. Patients infected with *Trypanosoma cruzi (T. cruzi)* were grouped as indeterminate (IND) and cardiac (CARD) forms ranging from 23 to 69 years of age (mean of 45.6±11.25). The IND group included 82 individuals, ranging from 24 to 66 years of age (mean of 39.6±10.3). The CARD group included 94 patients ranging from 23 to 69 years of age (mean of 48±12.52) presenting dilated cardiomyopathy. None of the patients have undergone chemotherapeutic treatment, nor had been previously treated for *T. cruzi* infection. Healthy non-chagasic individuals, ranging from 29 to 55 years of age (mean of 42.6±8.8) were included as a control group (NI). IND patients have a higher intensity of interleukin 10 (IL-10) expression when compared with individuals in the other groups. By contrast, inflammatory cytokine expression, such as interferon gamma (IFN-γ), tumor necrosis factor alpha (TNF-α), interleukin 6 (IL-6), and interleukin 1 beta (IL-1β), proved to be the highest in the CARD group. Correlation analysis showed that higher IL-10 expression was associated with better cardiac function, as determined by left ventricular ejection fraction and left ventricular diastolic diameter values. Altogether, these findings reinforce the concept that a fine balance between regulatory and inflammatory cytokines represents a key element in the establishment of distinct forms of chronic Chagas disease.

## Introduction

Chronic cardiomyopathy represents the most important and severe manifestation of human Chagas disease, eventually affecting approximately 20–30% of those in the chronic phase of the disease. The majority of the chronically affected individuals present the indeterminate (IND) form of the disease, with an apparent absence of morbidity [1], [2]. Epidemiological studies in endemic areas have shown that 2–5% of patients will evolve each year from the indeterminate to a clinical form of the disease [3]

A plethora of data has demonstrated that the host's immune response plays a key role in the differential clinical evolution of the disease. However, the mechanisms involved on the development of severe forms of Chagas disease are not well understood. While the balance between inflammatory and anti-inflammatory cytokines produced by circulating cells in patients with IND form leans towards to an anti-inflammatory profile, patients with chagasic cardiomyopathy seems to display a predominantly inflammatory environment [4]–[8].

Prior results have demonstrated that monocytes from both IND and CARD are able to produce IL-10. Furthermore, monocytes from IND patients produce higher levels of IL-10 than those from CARD patients [4], [5], [7]. However, CARD patients have an increased number of IFN-γ-producing CD8^+^ T cells, with reduced numbers of cells secreting IL-10 and lower percentages of FOXP3^+^-regulatory T cells [5]–[6], [9]–[13] when compared to IND patients. Nevertheless, the studies in this field have usually employed small samples of patients and statistical analyses that do not consider the wide dispersion of cytokine production observed in these patients. The aim of this study was to evaluate the plasma cytokine levels in well-defined clinical polar groups of chagasic patients divided into categories that better reflect the wide cytokine profile and its relationship with morbidity.

## Materials and Methods

### Study population

This study employed a cross-sectional design involving patients from endemic areas within the state of Minas Gerais, Brazil, under the medical care of one of the authors of this study (MOCR). The patients who agreed to participate in this study were volunteers who had been identified and selected at the Referral Outpatient Center for Chagas Disease at the Clinical Hospital of the Federal University of Minas Gerais (UFMG), Brazil. Positive serology for Chagas disease was determined by two or more tests (indirect immunofluorescence, enzyme-linked immunosorbent assay [ELISA], or indirect hemagglutination). Patients who agreed to participate in this study signed a written informed consent form and were subjected to a standard screening protocol that included medical history, physical examination, electrocardiogram (EKG), laboratory and chest X-ray examinations, and echodopplercardiography evolution. None of the patients were undergoing chemotherapeutic treatment nor had been previously treated for *T. cruzi* infection.

Individuals with any other chronic inflammatory diseases, thyroid dysfunction, valvular heart disease, coronary artery disease, systemic arterial hypertension, chronic obstructive pulmonary disease, hydroeletrolytic disorders, renal insufficiency, diabetes mellitus, alcoholism, and other infectious diseases were excluded from this study. A total of 176 patients with positive specific serology for *T. cruzi*, in the chronic phase of the disease and with well-defined clinical classification, were enrolled in this study. In addition, 24 healthy individuals were also included as the control group. Patients infected with *T. cruzi* were grouped as indeterminate (IND) or with cardiomyopathy (CARD). The IND group included 82 asymptomatic individuals, with ages ranging from 24 to 66 years (mean of 39.6±10.3), with no significant alterations in electrocardiography, chest X-ray, and echocardiogram. The CARD group included 94 patients, with ages ranging from 23 to 69 years (mean of 48±12.52), who presented dilated cardiomyopathy, characterized by the echocardiographic finding of a dilated left ventricle with impaired ventricular systolic function. LVEF and LVDD were used as the clinical parameters of the ventricular function for Chagas disease patients, where LVEF <55% and LVDD/body surface area ≥31 mm were used to define Chagas dilated cardiomyopathy [2]. None of the patients had undergone chemotherapeutic treatment nor had been previously treated for *T. cruzi* infection. Healthy individuals, ranging from 29 to 55 years of age (mean of 42.6±8.8), from a non-endemic area for Chagas disease and showing negative serological tests for the infection were included as a control group (non-infected [NI]).

### Ethics statement

Informed written consent was obtained from all individuals prior to their inclusion in the study. Treatment and clinical care were offered to all patients, as needed, regardless of whether or not the individual was enrolled in this research project. This study was carried out in full accordance with all international and Brazilian accepted guidelines and was approved by the Ethics Committee at René Rachou Research Center – FIOCRUZ (14/2006 CEPSH-IRR) and UFMG under protocol COEP-ETHIC 502/11).

### Blood samples

A 5-ml sample of peripheral blood was collected by venipuncture from each subject using a sterile Vacuntainer flask containing ethylenediamine tetraacetic acid (EDTA) as an anticoagulant. The samples were collected by a trained nurse at the Referral Center for Chagas Disease, Minas Gerais, Brazil. After the collection, whole peripheral blood was analyzed by flow cytometry using pre-defined parameters as previously established by our group [10].

### Detection of cytokine levels in plasma by Cytometric Bead Array (CBA)

Both a CBA Human Th1/Th2/Th17 Kit and a CBA Human Inflammatory Cytokine Kit (BD Biosciences, USA) were used to measure cytokine levels in plasma, following manufacturer instructions and that described in prior studies [10], [14]. The data were acquired in a Becton Dickinson FACScalibur flow cytometer and analyses performed using BD CBA software (BD Biosciences, USA). The following cytokines were measured: IL-10, IFN-γ, TNF-α, IL-6, and IL-1β. The results are expressed by mean intensity of fluorescence (MIF).

### Statistical analyses

Statistical comparative analyses were performed in groups of two between the NI, IND, and CARD groups, using the non-parametric Kruskal-Wallis test and Mann-Whitney U test, together with the Bonferroni correction (significance level, 0.05/3 = 0.0167). To investigate the relationship among immunological markers and cardiac function variables, Spearman correlation coefficients were calculated.

Statistical analyses were performed and graphs constructed using the R 2.15.0 software. All tests were performed considering a significance level of 5% (α = 0.05).

## Results

### Different plasma cytokine levels can be associated with cardiac morbidity in Chagas disease

The expressions of different cytokines (IL-10, IFN-γ, TNF-α, IL-6, IL-1β) were evaluated in the plasma of healthy individuals and patients with different forms of Chagas disease. The three groups were compared in groups of two for their cytokine expressions. The hypothesis of equality between the groups was discarded. Differences among the three groups could be observed for all evaluated cytokines (*P*<0.0001), with the exception of IL-1β.

**Figure 1:**
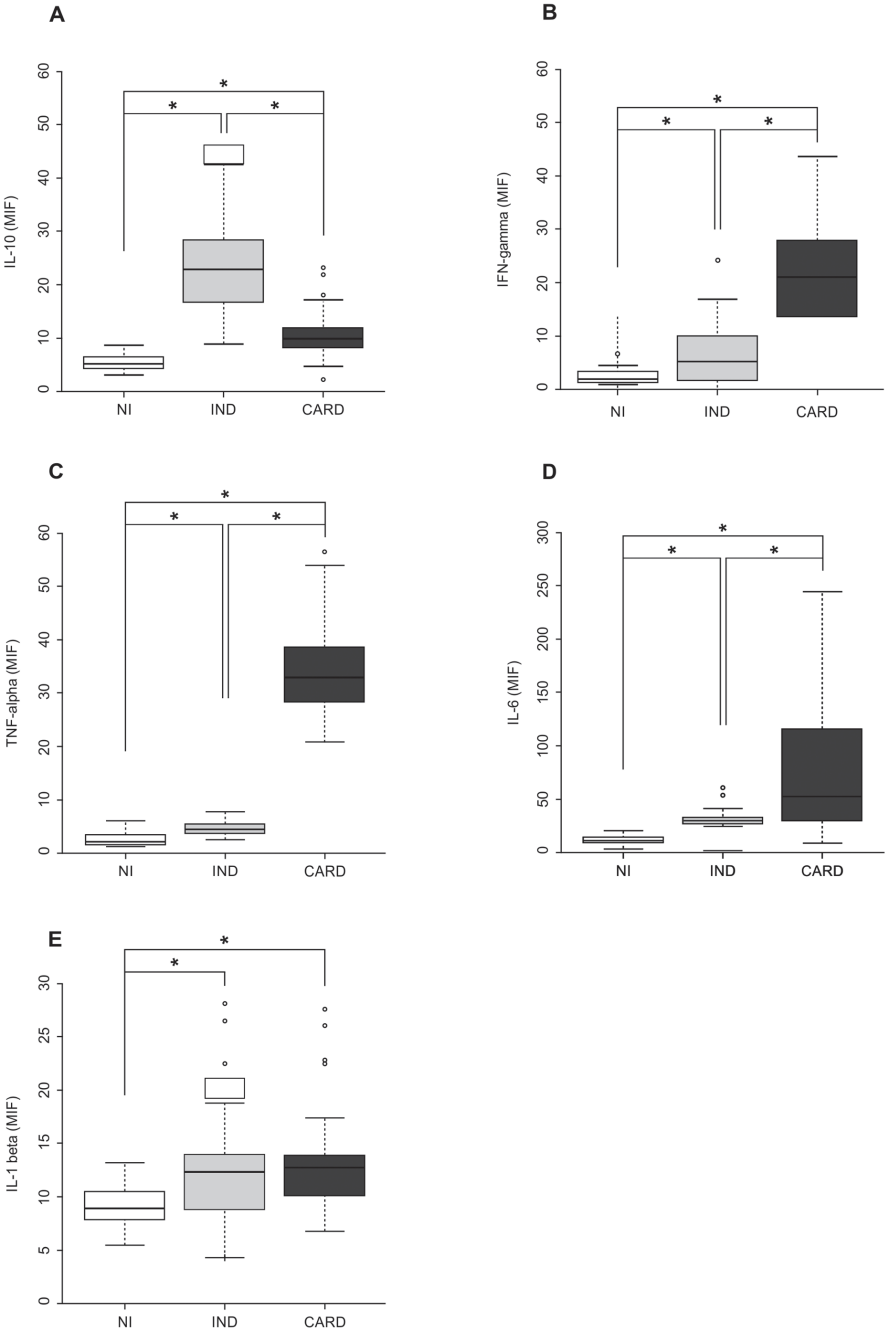
Analyses of plasma cytokine levels and their association with cardiac morbidity expressed by the clinical classification. The analysis of plasma levels was performed as described in the material and methods section. The groups evaluated were: NI (n = 24, white box), IND (n = 82, light gray box), and CARD (n = 94, dark gray box). The results were expressed by mean intensity of fluorescence (MIF). (A) Plasma IL-10 levels in NI, IND, and CARD groups and their association with cardiac morbidity. (B) Plasma IFN-γ levels in NI, IND, and CARD groups and their association with cardiac morbidity. (C) Plasma TNF-α levels in NI, IND, and CARD groups and their association with cardiac morbidity. (D) Plasma IL-6 levels in NI, IND, and CARD groups and their association with cardiac morbidity. E) Plasma IL-1β levels in NI, IND, and CARD groups and their association with cardiac morbidity. Significant differences (*P*-value<0.05) in the charts are identified by connecting lines and the symbol (*) for comparisons between the groups.

Data analysis showed that the IND group presented significantly higher levels of IL-10, median of 22.91 (8.98–42.49), as compared to the CARD group, median of 9.93 (2.31–25.58) and the NI group, median of 5.26 (3.19–8.77) (*P*<0.0001) (Figure 1A). In contrast, patients from the CARD group, when compared to the patients from the other groups, presented significantly higher levels of inflammatory cytokines, such as IFN-γ (Figure 1B), TNF-α (Figure 1C), and IL-6 (Figure 1D). A difference in the profile of these inflammatory cytokines could be observed when comparing the CARD and IND groups (*P*<0.0001), the CARD and NI groups (*P*<0.0001), and the IND and NI groups (*P*<0.0013), where significantly lower levels of inflammatory cytokines could be observed in the NI group when compared to the other groups. However, no significant difference could be observed when comparing the IL-6 expression in the IND and NI groups (*P* = 0.061) (Figure 1D). Furthermore, analysis of IL-1β expression showed no significant difference between the CARD and IND groups (*P* = 0.195) (Figure 1E). In the CARD group, the expression of IL-6 and TNF-α was significantly higher (medians 50.85 and 31.76, respectively) than IFN-γ (median 17.44) and IL-1β (median 12.58) (*P*<0.0001). This finding clearly shows that IL-10 is higher in the IND group, while IFN-γ, TNF-α, and IL-6 are higher in the CARD groups, as compared to the NI group.

**Figure 2:**
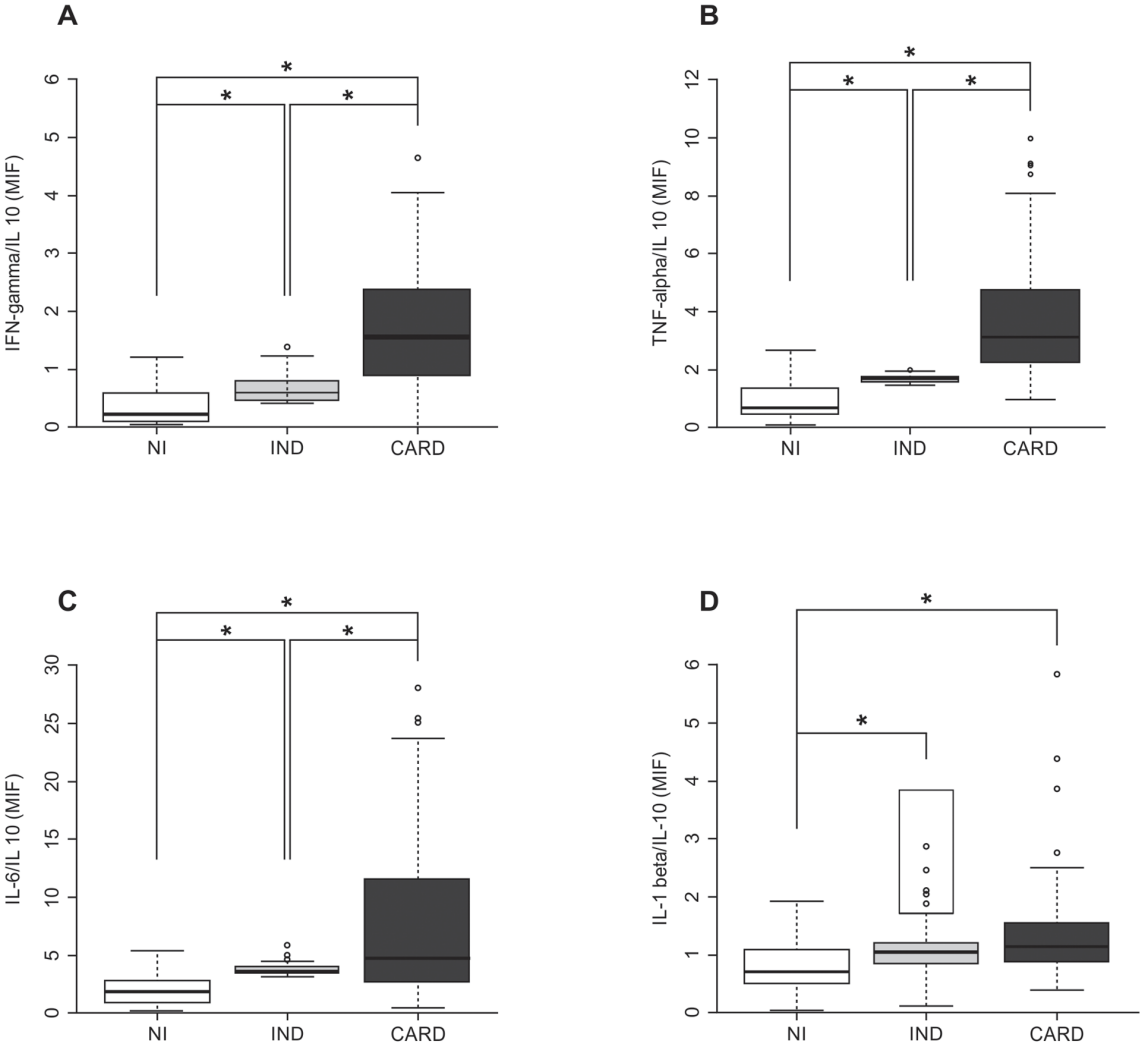
Ratios of plasma cytokines from patients with distinct forms of Chagas disease. The analysis of plasma levels was performed as described in the material and methods section. The groups evaluated were: NI (n = 24, white box), IND (n = 82, light gray box), and CARD (n = 94, dark gray box). The results are expressed by mean intensity of fluorescence (MIF). A) Ratios between IFN-γ and IL-10 levels in NI, IND, and CARD groups. B) Ratios between TNF-α and IL10 levels in NI, IND, and CARD groups. C) Ratios between IL-6 and IL-10 levels in NI, IND, and CARD groups. D) Ratios between IL-1β and IL-10 levels in NI, IND, and CARD groups. This analysis was employed to determine the balance between high-inflammatory or high-regulatory cytokines and to represent the predominant cytokine profile. Significant differences (*P*-value<0.05) in the charts are identified by connecting lines and the symbol (*) for comparisons between the groups.

IFN-γ/IL-10 and TNF-α/IL-10 ratios proved to be significantly higher in the CARD patients (*P*<0.0001) than in the IND and NI patients. (Figure 2A and Figure 2B, respectively). IL-6/IL-10 and IL-1β/IL-10 ratios were significantly higher in the CARD group (*P*<0.0001) than in the others (Figure 2C and Figure 2D, respectively).

### Establishing the cut-off to define low, medium, and high cytokine producers

**Figure 3:**
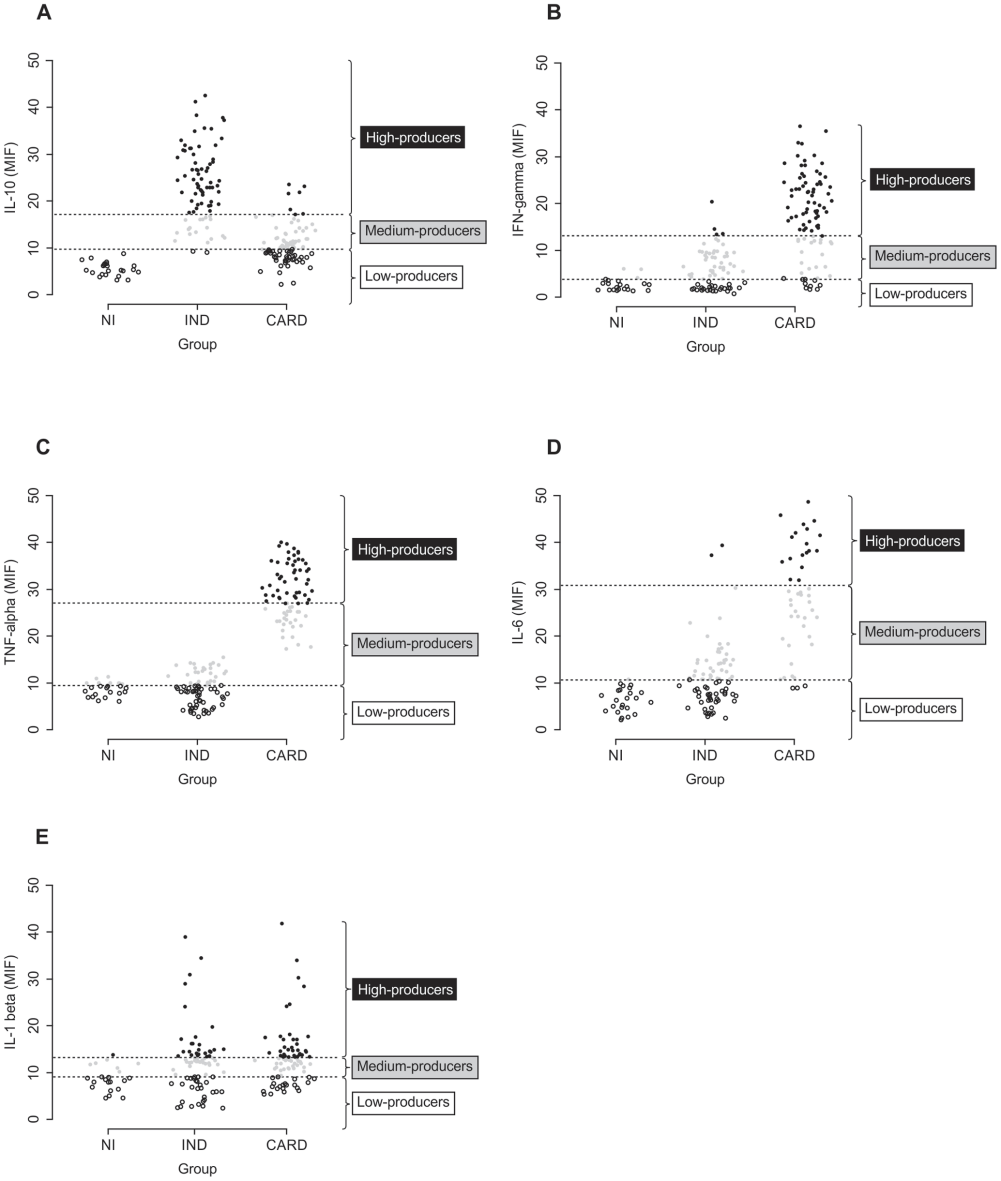
Establishing the concept of low, medium and high cytokine producers. A) Representative scatter plot graph of plasma IL-10 used to establish the cut-off to define low, medium, and high cytokine producers. B) Representative scatter plot graph of plasma IFN-γ used to establish the cut-off edge to define low, medium, and high cytokine producers. C) Representative scatter plot graph of plasma TNF-α used to establish the cut-off to define low, medium, and high cytokine producers. D) Representative scatter plot graph of plasma IL-6 used to establish the cut-off to define low, medium, and high cytokine producers. E) Representative scatter plot graph of plasma IL-1β used to establish the cut-off to define low, medium, and high cytokine producers. Low cytokine producers were defined by values of lower than the first tertile. Medium cytokine producers were defined by values equal to or lower than the second tertile, while high cytokine producers were defined by values higher than or equal to the second tertile. Results were considered significant with a *P*-value<0.05.

To establish the different cytokine cut-off that would allow for the identification of patients with polar forms of Chagas disease, an analysis was performed that allows a division of these individuals into three categories: low, medium, and high cytokine producers. This division was based on the detailed analysis of individual levels of cytokine expression obtained for NI, IND, and CARD groups; a statistical method was employed to determine the cut-off for tertiles, as shown in Figure 3 for IL-10, IFN-γ, TNF-α, IL-6 and IL-1β plasma levels. This method reflects the wide dispersion and non-parametric distribution of cytokine expression, as occurs in chronic Chagas cardiopathy.

The data analysis demonstrated that the IND group contains a significantly higher frequency (*P*<0.001) of high IL-10 producers (76% 63/82) than do the other groups (Figure 3A). Additional analysis revealed that a small proportion (7.5%, 7/94) of CARD patients displayed IL-10 plasma values of above the cut-off for IL-10 high cytokine producers (Figure 3A). Moreover, none of the individuals could be identified as high IL-10 producers in the NI group (Figure 3A). By contrast, most of the CARD patients displayed values of IFN-γ plasma that are above the cut-off, being significantly higher than NI and IND individuals (*P*<0.001), while approximately 9.6% (9/94) and 21.3% (20/94) of CARD patients were identified as low and medium IFN- producers, respectively (Figure 3B). It is interesting to note that the analysis of plasma cytokines demonstrated that none of the IND individuals fell into a region of high TNF-α producers, unlike CARD patients who were confined to a level above the cut-off established for high TNF-α producers (Figure 3C). In the CARD group, it could be observed that most of the IL-6 producers tended to be confined to the second tertile, established as the cut-off for medium IL-6 producers (26.6%, 25/94), while the frequency of high IL-6 producers (19.15%, 18/94) was higher than that found for low IL-6 producers from this same group (3.2%, 3/94) (*P*<0.001) (Figure 3D). Furthermore, no significant difference in the frequency of high-IL-1β producers could be observed when comparing the CARD (30.5%, 25/82) and IND (36.17%, 34/94) groups (*P = *0.171) (Figure 3E).

### Distinct plasma cytokine levels and echocardiographic variables correlate with different chagasic cardiac morbidity

To confirm whether higher levels of IL-10 in fact correlates with improved cardiac function in human Chagas disease, correlation analysis was performed between the plasma levels of IL-10 in either CARD or IND producers and cardiac performance expressed by two clinical variables: LVEF and LVDD. Similarly, correlation analysis was performed to determine how inflammatory cytokines (IFN-γ, TNF-α, IL-6, and IL-1β) are associated with cardiac function.

**Figure 4:**
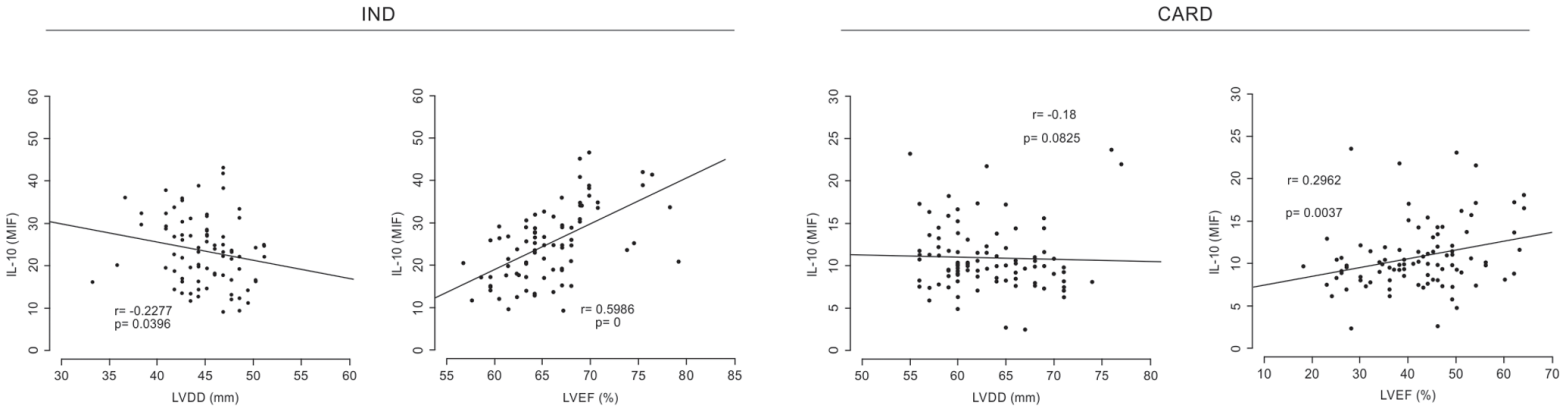
Correlation analysis between plasma IL-10 levels and echocardiographic variable markers of cardiac morbidity. Correlation analysis between plasma IL-10 levels and cardiac function variables (LVEF and LVDD) in the IND (n = 82, first column) and CARD (n = 94, second column) groups. Correlation analysis was performed using the Spearman correlation coefficient, and results were considered significant when *P*<0.05. Significant differences (*P*-value) are indicated in each graph together with the *r* value.

As expected, a significant negative correlation between lower LVDD and higher levels of IL-10 could be observed in the IND group (Figure 4A). However, a significant positive correlation could be found between higher LVEF and higher levels of IL-10 in the same group (Figure 4A). No significant correlation was identified when IL-10 levels were compared with LVDD in the CARD group (Figure 4A). Positive correlation was observed between higher LVEF and higher levels of IL-10 in CARD group (Figure 4A).

**Figure 5:**
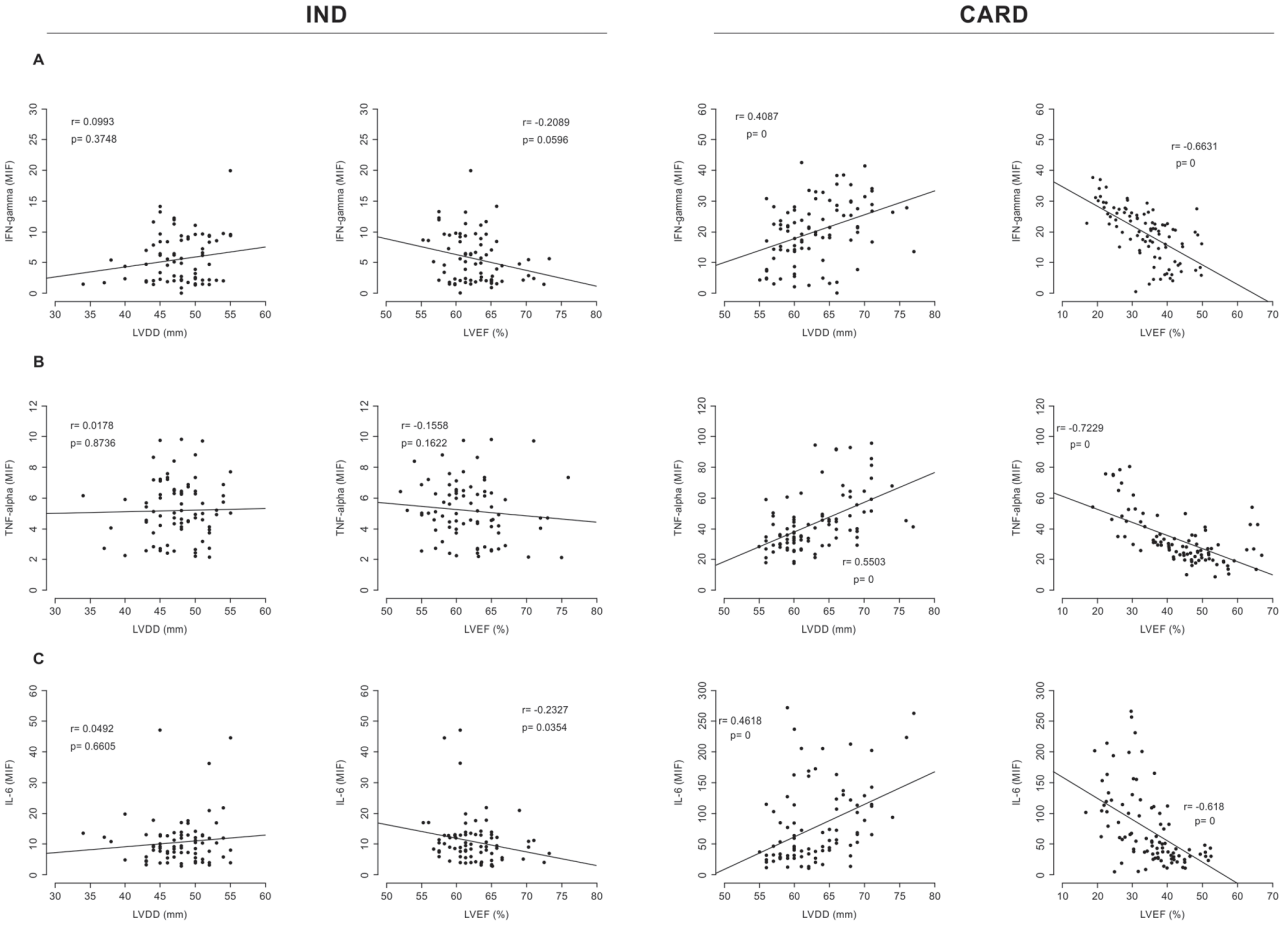
Correlation analysis between plasma IFN-γ, TNF-α, IL-6 levels, and echocardiographic variable markers of cardiac morbidity. A) Correlation analysis between plasma IFN-γ and cardiac function variables in the IND (n = 82, first column) and CARD (n = 94, second column) groups. B) Correlation analysis between plasma TNF-α and cardiac function variables in the IND (n = 82, first column) and CARD (n = 94, second column) groups. C) Correlation analysis between plasma IL-6 and cardiac function variables in the IND (n = 82, first column) and CARD (n = 94, second column) groups. Correlation analysis was performed using the Spearman correlation coefficient, and results were considered significant when *P*<0.05. Significant differences (*P-*value) are indicated in each graph together with the *r* value.

No correlation between higher LVDD and higher levels of IFN-γ were identified in the IND group (Figure 5A). However, a significant negative correlation between lower LVEF and higher levels of IFN-γ was observed in the same group, although not as significant (Figure 5A). A significant positive correlation was observed between higher LVDD and higher levels of IFN-γ in patients with cardiomyopathy (Figure 5A). Conversely, a significant negative correlation was observed between lower LVEF and higher levels of IFN-γ in the CARD group (Figure 5A). Comparative analysis of TNF-α levels and LVDD in the CARD group, showed a positive correlation (Figure 5B). Surprisingly, in cardiomyopathy patients, TNF-α levels were inversely correlated with LVEF (Figure 5B). No correlation was observed between TNF-α levels and echocardiographic variables of the cardiac function in the IND group (Figure 5B). Similar results were observed for IL-6 levels (Figure 5C). Correlation analysis between higher IL-6 levels and higher LVDD in the CARD group showed a significant positive correlation (Figure 5C). In contrast, a significant negative correlation was observed between IL-6 levels and LVEF in same group. Interestingly, in all studied groups, no significant correlations were observed when IL-1β levels and LVDD (IND group, *r* = 0.1205, *P* = 0.2809 and CARD group *r* = 0.0372, *P* = 0.7217) and LVEF (IND group, *r* = −0.0707, *P* = 0.4985 and CARD group *r* = 0.0707, *P* = 0.7217) were evaluated.

## Discussion

The exact mechanisms associated with the development of pathology in human Chagas disease have yet to be fully clarified. It has been hypothesized that alterations in the immune response are more likely to be involved in the pathogenesis of Chagas disease and that the chronic forms result from multifactorial immunological mechanisms. An impaired cytokine network has been pointed out as one of the determining factors in disease morbidity [5], [8], [15].

The present work revealed that CARD patients, when compared to IND and NI individuals, present a strong immune response expressed by higher levels of IFN-γ, TNF-α, and IL-6. In contrast, higher levels of IL-10 could be observed in the IND group when compared to the NI and CARD groups. The fact that all plasma cytokine levels evaluated in the IND group were higher, when compared to the NI group, may reflect the effect of an ongoing infection in these patients. This infection may continue to signal cytokine secretion even if it occurs at much lower levels. This is certainly expected and therefore not a surprising finding when compared to healthy controls. These results reinforce findings from previous studies [4]–[5], [8]–[10], [16]–[21], and the methodology used herein allows for a more accurate interpretation of the cytokine expression pattern and its relationship with morbidity in Chagas disease.

Interestingly, a wide variation could be observed in the groups evaluated for their cytokine expression. This variability was observed when the plasma levels of cytokines in each group of patients of the study were analyzed. Despite the significant variability observed in the cytokine levels and an inflammatory phenotype, a direct association with the severe forms of this disease could be identified. Conversely, different levels of inflammatory cytokines were inversely associated with individuals classified with the IND form of Chagas disease. Similarly, a direct association could be observed between high levels of IL-10, which presented an anti-inflammatory activity, and the IND form. The ratios between inflammatory and regulatory cytokines, as shown in the present study, were significantly higher in the CARD group than that verified in the IND and NI groups. Analysis of these findings showed a clear relationship between cytokine expression and cardiac morbidity as determined by clinical classification of the individuals. It has been postulated that modulation of the activity of macrophages and Treg cells producing IL-10 may maintain the balance between parasitism and tissue integrity in IND patients [5]. In addition, a contained Th1 response would perhaps keep parasitism under relative control. The progressive destructive process in patients with cardiomyopathy could therefore be the result of a decrease in the regulatory activity of the pathogenic Th1 response due to a decrease in IL-10 production. This decrease may well be associated with the host's genetic characteristics, which are age-dependent, or may be affected by unrelated infections caused by *T. cruzi* reinfection or by other microorganisms [6].

Prior literature has already reported that a detailed analysis of individual levels of cytokines allows one to characterize the profile of NI, IND and CARD individuals [5], [8], [16]. In these studies, when IND and CARD patients were classified according to the amounts of IFN-γ produced by peripheral blood mononuclear cell (PBMC) cultures stimulated with *T. cruzi* antigens as high (>5.0 ng/ml) and low (<4.9 ng/ml) producers; the cutoff corresponded to the mean value of unstimulated control cultures added with two values of standard deviation [5], [16]. Moreover, even the global median percentage of each cell population expressing IFN-γ, IL-4, IL-10, IL-12, IL-13, and TNF-α [8] was used to establish the cut-off to divide the individuals into two categories: low and high producers. The present study employed an alternative strategy in an attempt to establish a more feasible and adequate determination of the cut-off that allows for a division of the individuals according to the cytokine production level. The chosen method was developed to more properly reflect the wide dispersion and non-parametric distribution of the cytokine expression that occurs in chronic chagasic cardiopathy. To establish a cut-off that allows for the division of the individuals into three categories – low, medium, and high cytokine producers – a specific statistical method based in tertiles was used to determine the cut-off points [22].

Sixty-nine percent of the CARD patients were defined as high IFN-γ producers, whereas only 4.9% of the IND patients presented this profile for IFN-γ levels. The CARD group showed 71.3% of high TNF-α producers, as compared to the IND group, which was distributed only among the low and medium TNF-α producers. Similar results could also be identified when analyzing the IL-6 levels in the evaluated groups, although to a lesser extent. These findings reinforce a relationship between the expression of these inflammatory cytokines, especially IFN-γ and TNF-α, and the development of heart disease. Previous reports have also shown that IFN-γ is the cytokine most frequently detected in the inflammatory cells of the myocardium of patients with Chagas disease [23]. It is currently accepted that IFN-γ production may contribute to the development of cardiomyopathy. The present study showed a small proportion of IND patients presenting high levels of IFN-γ, whereas a significant percentage of these patients expressed high levels of IL-10. A recent study on the functional polymorphism 251082 G/A of the IL-10 gene revealed that the polymorphic allele, related to the lower IL-10 expression, is associated with the development of chagasic cardiomyopathy. This finding suggests that the expression of IL-10 is indeed an important factor in the modulation of the inflammatory response and that it can be genetically determined [24].

Correlation analysis was performed to compare plasma cytokine levels and echocardiographic variables of the cardiac function (LVEF and LVDD). These distinct clinical variables are directly and inversely correlated with a better cardiac function, respectively [2]. A significant positive correlation between IL-10 levels and LVEF in the IND group could be observed. In these patients, no correlation could be identified between the expression of this cytokine and LVDD. Conversely, a significant and strong inverse correlation between plasma inflammatory cytokine levels (IFN-γ, TNF-α, and IL-6) and LVEF was observed in the CARD group. In contrast, only IFN-γ and TNF-α presented a significant direct correlation, albeit less expressive with LVDD in this same group. These results suggest that IL-10 plays an important regulatory role in Chagas disease and is clearly involved in protecting against cardiac damage, while inflammatory cytokines, such as IFN-γ and TNF-α, seem to be associated with the development of heart disease in this infection.

The data obtained in this paper contributes to the understanding of the physiopathology of Chagas disease due to some of its methodological characteristics. First, the large, well-defined samples of chagasic patients who were not undergoing chemotherapeutic treatment, nor had been previously treated for *T. cruzi* infection, and the analysis of cytokine plasma levels in three categories, more faithfully expressing the wide dispersion observed in those patients within natural conditions, provide a greater reliability to these results. Second, cytokine data from a very large and homogenous cohort of indeterminate and cardiac patients, as well as from non-infected controls, led to clear-cut conclusions. Third, the comparison of cytokine data with echocardiographic variables of cardiac function, such as LVEF and LVDD, sparks interest and adds credibility to the observed correlations.

Within the broad distribution ranges of cytokine levels produced by the IND and CARD patients, patients characterized by the production of low and high levels of regulatory and inflammatory cytokines can be observed in both groups. The significance of this differential expression as a possible determining factor of disease progression is unknown. It is possible that IND patients with higher levels of inflammatory cytokines may well be those that are more likely to develop cardiac forms of the disease, while those that show higher levels of regulatory cytokines tend to remain asymptomatic. By contrast, CARD patients with higher levels of inflammatory cytokines would be those who are prone to present an earlier and more severe progression toward cardiac involvement, whereas those showing higher levels of regulatory cytokines and lower levels of inflammatory cytokines would present later and less intense heart disease. These assumptions involve important implications to better understand the pathogenesis, natural history, and clinical management of patients with Chagas disease, thus warranting further investigation.

Taken together, the findings of this study reinforce the concept that a fine balance between regulatory and inflammatory cytokines represents a key element in the establishment of the distinct forms of chronic Chagas disease.
